# Copy number variants and their implications for developmental and behavioural problems in cleft lip and/or palate

**DOI:** 10.1093/hmg/ddaf115

**Published:** 2025-07-15

**Authors:** Alexandros Rammos, Rachel Blakey, Charlotte A Dennison, Sarah J Lewis, Nabila Ali, Amy Davies, Yvonne Wren, Kerry Humphries, Jonathan Sandy, Elliott Rees, Kimberley Marie Kendall, Gemma C Sharp, Michael J Owen, Marianne B M van den Bree, Evie Stergiakouli

**Affiliations:** Cleft Collective, University of Bristol, Oakfield House, Oakfield Grove, Bristol BS8 2BN, United Kingdom; Population Health Sciences, Bristol Medical School, Oakfield House, Oakfield Grove, Bristol BS8 2BN, United Kingdom; MRC Integrative Epidemiology Unit, University of Bristol, Oakfield House, Oakfield Grove, Bristol BS8 2BN, United Kingdom; Cleft Collective, University of Bristol, Oakfield House, Oakfield Grove, Bristol BS8 2BN, United Kingdom; Population Health Sciences, Bristol Medical School, Oakfield House, Oakfield Grove, Bristol BS8 2BN, United Kingdom; MRC Integrative Epidemiology Unit, University of Bristol, Oakfield House, Oakfield Grove, Bristol BS8 2BN, United Kingdom; Wolfson Centre for Young People's Mental Health, Hadyn Ellis Building, Maindy Road, Cardiff University, Cardiff CF24 1TP, United Kingdom; The Centre for Neuropsychiatric Genetics and Genomics, Division of Psychological Medicine and Clinical Neurosciences, Cardiff University, Hadyn Ellis Building, Maindy Road, Cardiff CF24 1TP, United Kingdom; Cleft Collective, University of Bristol, Oakfield House, Oakfield Grove, Bristol BS8 2BN, United Kingdom; Population Health Sciences, Bristol Medical School, Oakfield House, Oakfield Grove, Bristol BS8 2BN, United Kingdom; MRC Integrative Epidemiology Unit, University of Bristol, Oakfield House, Oakfield Grove, Bristol BS8 2BN, United Kingdom; The Centre for Neuropsychiatric Genetics and Genomics, Division of Psychological Medicine and Clinical Neurosciences, Cardiff University, Hadyn Ellis Building, Maindy Road, Cardiff CF24 1TP, United Kingdom; Neuroscience and Mental Health Innovation Institute, Cardiff University, Hadyn Ellis Building, Maindy Road, Cardiff CF24 1TP, United Kingdom; School of Psychology, University of Leeds and Born in Bradford’s Centre for Applied Education Research, Bradford Royal Infirmary, Duckworth Lane, Bradford BD9 6RJ, United Kingdom; Cleft Collective, University of Bristol, Oakfield House, Oakfield Grove, Bristol BS8 2BN, United Kingdom; Bristol Dental School, University of Bristol, 1 Trinity Quay, Avon Street, Bristol BS2 0PT, United Kingdom; Cleft Collective, University of Bristol, Oakfield House, Oakfield Grove, Bristol BS8 2BN, United Kingdom; Bristol Dental School, University of Bristol, 1 Trinity Quay, Avon Street, Bristol BS2 0PT, United Kingdom; Centre for Speech, Hearing and Communication Research, Cardiff Metropolitan University, Llandaff Campus, 200 Western Avenue, Cardiff CF5 2YB, United Kingdom; Cleft Collective, University of Bristol, Oakfield House, Oakfield Grove, Bristol BS8 2BN, United Kingdom; Bristol Dental School, University of Bristol, 1 Trinity Quay, Avon Street, Bristol BS2 0PT, United Kingdom; Cleft Collective, University of Bristol, Oakfield House, Oakfield Grove, Bristol BS8 2BN, United Kingdom; The Centre for Neuropsychiatric Genetics and Genomics, Division of Psychological Medicine and Clinical Neurosciences, Cardiff University, Hadyn Ellis Building, Maindy Road, Cardiff CF24 1TP, United Kingdom; Neuroscience and Mental Health Innovation Institute, Cardiff University, Hadyn Ellis Building, Maindy Road, Cardiff CF24 1TP, United Kingdom; The Centre for Neuropsychiatric Genetics and Genomics, Division of Psychological Medicine and Clinical Neurosciences, Cardiff University, Hadyn Ellis Building, Maindy Road, Cardiff CF24 1TP, United Kingdom; School of Psychology, University of Leeds and Born in Bradford’s Centre for Applied Education Research, Bradford Royal Infirmary, Duckworth Lane, Bradford BD9 6RJ, United Kingdom; Department of Psychology, Faculty of Health and Life Sciences, University of Exeter, St Luke's Campus, Heavitree Road, Exeter EX4 4PY, United Kingdom; The Centre for Neuropsychiatric Genetics and Genomics, Division of Psychological Medicine and Clinical Neurosciences, Cardiff University, Hadyn Ellis Building, Maindy Road, Cardiff CF24 1TP, United Kingdom; Neuroscience and Mental Health Innovation Institute, Cardiff University, Hadyn Ellis Building, Maindy Road, Cardiff CF24 1TP, United Kingdom; The Centre for Neuropsychiatric Genetics and Genomics, Division of Psychological Medicine and Clinical Neurosciences, Cardiff University, Hadyn Ellis Building, Maindy Road, Cardiff CF24 1TP, United Kingdom; Neuroscience and Mental Health Innovation Institute, Cardiff University, Hadyn Ellis Building, Maindy Road, Cardiff CF24 1TP, United Kingdom; Cleft Collective, University of Bristol, Oakfield House, Oakfield Grove, Bristol BS8 2BN, United Kingdom; Population Health Sciences, Bristol Medical School, Oakfield House, Oakfield Grove, Bristol BS8 2BN, United Kingdom; MRC Integrative Epidemiology Unit, University of Bristol, Oakfield House, Oakfield Grove, Bristol BS8 2BN, United Kingdom

**Keywords:** copy number variants, cleft collective, longitudinal outcomes, behavioural problems, cleft lip and/or palate

## Abstract

Cleft lip and/or palate (CL/P) is the most common craniofacial congenital anomaly and has been associated with higher risk of neurodevelopmental and behavioural problems indicating potential shared genetic factors between CL/P and neurodevelopmental disorders. In this study, we aimed to determine the prevalence of neurodevelopmental copy number variants (CNV) in children with CL/P and their link to early developmental and behavioural problems. Using data from the Cleft Collective, the largest UK-based national cohort study of children with CL/P, we determined the rates of neurodevelopmental CNVs in children with CL/P comparing them to the general population, explored differences by cleft type and investigated risk of developmental delays and behavioural problems among those with CL/P and neurodevelopmental CNVs. Children with CL/P had a higher prevalence of neurodevelopmental CNVs than participants in four population-based samples (3.7% vs 2.3% in the Avon Longitudinal Study of Parents and Children (ALSPAC), 2.0% in Born in Bradford (BiB), 2.3% in Millenium Cohort Study (MCS), 1.7% in UK Biobank, ORs(95%CIs): ALSPAC = 1.56(1.18–2.06), BiB = 1.84(1.37–2.45), MCS = 1.59(1.19–2.11), UK Biobank = 2.15(1.68–2.71). Children with cleft palate only were 3 times more likely to have a neurodevelopmental CNV (95%CIs1.50–6.59, p = 0.03) than children with cleft lip only. Furthermore, children with CL/P and neurodevelopmental CNVs were more likely to experience early developmental delays and behavioural problems by age 5 compared to children with CL/P and without neurodevelopmental CNVs. These findings highlight that genetic testing ascertaining the presence of neurodevelopmental CNVs might be helpful in early identification of developmental needs in children with CL/P.

## Introduction

Cleft lip and/or palate (CL/P) is the most common craniofacial congenital anomaly affecting one in every 1000 live births [1]. An estimated 30% of children born with CL/P have a syndromic form of cleft while for the remaining 70%, CL/P is considered non-syndromic. Syndromic CL/P usually presents with additional clinical phenotypes ranging from cognitive impairment to motor difficulties [2] and is caused by distinct genetic mutations or chromosomal differences [1]. Similar to other multifactorial phenotypes, including neurodevelopmental and behaviouralproblems, non-syndromic CL/P has a complex aetiology with both genetic and environmental factors implicated in the causal pathways to phenotypes [1, 3].

The most common types of CL/P are known as cleft lip (CL), cleft lip and palate (CLP), cleft palate only (CP) and submucous cleft palate (where some of the soft, but not hard, palate fails to fuse properly). Cleft palate has long been considered aetiologically different to other types of cleft, with different genetic loci implicated from genetic [4] and epigenetic [5] studies as well as higher rates of syndromic forms [6].

Some studies have reported higher rates of neurodevelopmental and behavioural problems in children born with CL/P compared to children from the general population [7–10], educational underachievement [11, 12]. A meta-analysis of behavioural problems in children born with CL/P reported that differences were mainly limited in higher rates of depression and anxiety symptoms [13]. However, this meta-analysis was conducted before larger recent investigations. While environmental factors secondary to CL/P such as surgeries, appearance differences and bullying might be contributing to the increased prevalence of some behavioural problems among children born with CL/P [14], it is possible that the higher prevalence of neurodevelopmental and behavioural problems among children born with CL/P is partially explained by genetic overlap between CL/P and neurodevelopmental disorders.

Amongst the genetic variants implicated in CL/P and in neurodevelopmental and behavioural problems, copy number variants (CNVs) have emerged as significant contributors to pathogenesis. CNVs are a collective term for duplications and deletions in segments of chromosomal DNA that are greater than 1 kilobase (kb) in size. Specific CNVs have been shown to be associated with CL/P [15–17], neurocognitive impairment [18, 19] and complex neuropsychiatric disorders [20–25]. Neurodevelopmental CNVs have not been systematically investigated in children born with CL/P, although there is some evidence of overlap between CNVs associated with CL/P and neurodevelopmental disorders, for example CP and developmental delay are both associated with 22q11 deletion syndrome [26]. It is possible that rates of neurodevelopmental CNVs are higher in children born with CL/P compared to children from the general population. Given the heterogeneity in genetic factors involved in different types of cleft and particularly CP, it is likely that higher rates of neurodevelopmental CNVs are observed in certain types of cleft. Neurodevelopmental CNVs in children born with CL/P could be also linked to the higher rates of early developmental concerns [10, 27] and behavioural problems observed in children born with CL/P [9].

We aim to determine the rate of neurodevelopmental CNVs among i) children born with any CL/P and ii) by specific cleft type, to describe which neurodevelopmental CNVs are present, and to test the following hypotheses:

(H1) Children born with CL/P have a higher rate of neurodevelopmental CNVs than the general population.

(H2) Rates of neurodevelopmental CNVs in children born with CL/P differ by cleft type.

(H3) Children born with CL/P and a neurodevelopmental CNV are at higher risk of developmental delay and behavioural problems than those born with CL/P and without a neurodevelopmental CNV. To assess early developmental problems in children born with CL/P we calculated mean developmental trajectories between 18 months to 5 years and compared them between children born with CL/P and a neurodevelopmental CNV and those born with CL/P but without a neurodevelopmental CNV. We also compared rates of behavioural problems between the two groups.

## Results

### Sample characteristics

The characteristics of the final sample of children born with CL/P from the Cleft Collective [28] who passed CNV Quality Control (QC) processes (N = 2180) are described in Table 1. The sample included more males (57%) than females (43%). Most participants had white mothers (91%), and 61% of all mothers had completed higher education (a degree or equivalent). Of those with genetic data for CNV calls, 998 (46%) had completed at least one neurodevelopmental or behavioural problem questionnaire during at least one-time point. A comparison of those with genetic data in the Cleft Collective to those without did not identify evidence of differences in terms of cleft type, syndromic status and mean scores in questionnaires assessing developmental delays and behavioral problems (Table S1).

**Table 1:** Sample characteristics of cleft collective participants. Sample is defined as those with genetic and phenotypic data after quality control. percentages calculated exclusive of missing data.

	With a neurodevelopmental CNV		Without a neurodevelopmental CNV	
	N (%)		N (%)	
Study Child	77		2103	
*Sex*				
Female	34 (44)		900 (43)	
Male	43 (56)		1203 (57)	
*Maternal ethnic group*				
White	37(48)		917(44)	
Other	7(9)		87(4)	
Missing	33(43)		1099 (52)	
*Maternal education*				
None/fewer than 5 GCSEs or equivalent	9(12)		108(5)	
Post-Secondary Education (Non-degree)	11(14)		253(12)	
Higher Education (Degree or equivalent)	23(30)		643(31)	
Missing	34(44)		1099 (52)	
*Cleft type*				
Cleft lip	9(12)		495(24)	
Cleft palate	41(53)		754(36)	
Cleft lip and palate	24(31)		763(36)	
Submucous cleft palate	0 (0)		22 (1)	
Missing	3 (4)		69(3)	
*Parent/surgeon reported syndrome*				
Yes	15 (19)		135 (6)	
No	52 (68)		1601 (76)	
Missing	10 (13)		367 (17)	
	**N**	**Mean (SD)**	**N**	**Mean (SD)**
*Age & Stages Questionnaire—third edition (ASQ-3)^1^*				
*ASQ-3 Communication*				
18 months	29	27.93 (17.35)	711	32.54 (16.33)
3 years	29	44.14 (18.76)	709	49.2 (14.79)
5 years	21	43.57 (19.69)	574	51.95 (14.51)
*ASQ-3 Fine Motor*				
18 months	29	46.38 (18.99)	711	51.67 (13.00)
3 years	29	35.00 (21.30)	709	43.84 (17.17)
5 years	21	37.86 (22.50)	574	49.00 (15.95)
*ASQ-3 Gross Motor*				
18 months	29	45.52 (15.77)	711	50.70 (15.25)
3 years	29	47.24 (17.76)	709	52.14 (13.79)
5 years	21	40.48 (19.87)	574	49.26 (14.91)
*ASQ-3 Personal Social*				
18 months	29	42.93 (12.85)	711	45.02 (12.77)
3 years	29	46.72 (12.34)	709	49.37 (14.18)
5 years	21	45.00 (16.05)	574	51.82 (12.76)
*ASQ-3 Problem Solving*				
18 months	29	35.69 (20.95)	711	39.28 (15.65)
3 years	29	43.97 (18.10)	709	51.85 (14.24)
5 years	21	45.95 (16.33)	574	52.77 (13.00)
*ASQ: Social Emotional –second edition* *(ASQ:SE-2)^2^*				
18 months	29	35.00 (25.70)	711	26.56 (24.27)
3 years	27	52.04 (57.72)	667	22.45 (32.87)
5 years	20	78.13 (77.34)	564	41.17 (48.32)
*Strengths & Difficulties Questionnaire (SDQ)^3^*				
Age 5				
*Emotional*	21	2.76 (2.55)	562	1.70 (1.93)
*Conduct*	21	2.95 (2.71)	562	1.75 (1.70)
*Hyperactivity*	20	5.00 (3.48)	561	4.14 (2.76)
*Peer Problems*	21	2.95 (2.06)	563	1.47 (1.88)
*Prosocial*	21	5.90 (2.93)	562	8.09 (2.20)
*Total Difficulties Score*	20	14.1 (8.30)	561	9.06 (6.39)

Abbreviations: CNVs, Copy Number Variants; GCSE, General Certificate of Secondary Education; ASQ-3, Age & Stages Questionnaire—third edition, ASQ: SE-2; Age & Stages Questionnaire: Social Emotional –second edition; Strengths and Difficulties Questionnaire. ^1^For ASQ-3 scores for each domain range from 0 to 60, with higher scores on the ASQ-3 domains indicating better developmental outcomes. ^2^For ASQ:SE-2 the possible score range for each age are as follows: 18 months; 0–360, 3 years; 0–465, 5 years; 0–405, with higher scores on the ASQ:SE-2 domains indicating worse developmental outcomes. ^3^Scores in the four subscales of the SDQ combine to form a total difficulties score, with a score of 17 or above flagging high risk. High-risk cut-offs for detailed assessment: conduct problems (≥ 4), emotional symptoms (≥ 5), hyperactivity (≥ 6), peer problems (≥ 7), and prosocial behaviour (< 4).

### Neurodevelopmental CNVs

We called a pre-determined list of 54 CNVs that have been previously associated with neurodevelopmental disorders [29] in children and parents from a UK-based national cohort study of children born with CL/P, the Cleft Collective, and compared their rates in four general population comparison groups; Avon Longitudinal Study of Parents and Children (ALSPAC) [30, 31], Born in Bradford (BiB) [32, 33], the Millenium Cohort Study (MCS) [34] and UK Biobank [35, 36] (see Methods for more details about the general population comparison groups used). The full list of CNVs used can be found in Table S2. After applying QC (described in Methods), we found strong evidence to support H1: Children born with any type of CL/P had a higher prevalence of neurodevelopmental CNVs than participants in all four general population comparison groups (3.7% vs 2.3% in ALSPAC, 2.0% in BiB, 2.3% in MCS, 1.7% in UK Biobank, ORs(95%CIs): ALSPAC = 1.56 (1.18–2.06), BiB = 1.84 (1.37–2.45), MCS = 1.59 (1.19–2.11), UK Biobank = 2.15 (1.68–2.71) (Table 2A and B). In addition, there was also strong evidence for H2: The prevalence of neurodevelopmental CNVs varied by cleft sub-type in children from the Cleft Collective (Table 2C). A total of 5.1% of children born with CP only had a neurodevelopmental CNV compared to 1.8% of children with cleft lip only. Children with CP were 2.98 times more likely to have a neurodevelopmental CNV (95%CIs = 1.50–6.59, p = 0.03) compared to children with cleft lip only and children from the general population comparison groups (ORs = 2.3–3.18, in all general population comparison groups) (Table 2C). We also note that children with a neurodevelopmental CNV were more likely to have a syndromic cleft as reported by a parent or their surgeon (OR = 3.42, 95%CIs; 1.74, 6.36, p = 2x10^−4^). However, 68% of those with neurodevelopmental CNVs did not report having a syndromic form of CL/P (Table 1).

**Table 2:** The frequency, percentage and odds ratios of children with neurodevelopmental disorder copy number variants (ND CNVs) among children with CL/P vs among general population cohorts and stratified by cleft type. Comparison cohorts are the Avon longitudinal study of parents and children (ALSPAC), born in Bradford (BiB), the Millenium cohort study (MCS) and UK biobank.

**A. CNV frequencies and rates in children born with CL/P, CL, CP, CLP from the Cleft Collective. ORs, 95% CIs and p values are for comparisons of CNV rates between children born with CP and CLP versus CL.**							
**Category**	**ND CNV+**	**ND CNV-**	**Total**	**Percentage**	**OR**	**95% CIs**	**p-value**
**CL/P**	77	2103	2180	3.70%	-	-	-
**Cleft Lip (CL)**	9	495	504	1.80%	ref	ref	ref
**Cleft Palate (CP)**	41	757	798	5.10%	2.98	1.50–6.59	0.004
**Cleft Lip and Palate (CLP)**	24	767	791	3.00%	1.72	0.79–3.73	0.17
**B. CNV frequencies and rates in participants from the ALSPAC, BiB, MCS and UK Biobank.**							
**Category**	**ND CNV+**	**ND CNV-**	**Total**	**Percentage**			
**ALSPAC**	144	6217	6361	2.30%			
**BiB**	149	7477	7626	2.00%			
**MCS**	151	6559	6710	2.30%			
**UK Biobank**	2583	1,49 036	1,51 619	1.70%			
**C. Comparisons of CNV rates between children born with CL/P, CL, CP, CLP from the Cleft Collective versus ALSPAC, BiB, MCS and UK Biobank**							
	**REFERENCE CATEGORIES**						
	**ALSPAC**	**BiB**	**MCS**	**UK Biobank**			
	**Cleft Collective CL/P**						
**OR**	1.56	1.84	1.59	2.15			
**95% CIs**	1.18–2.06	1.37–2.45	1.19–2.11	1.68–2.71			
**p-value**	0.002	3.9x10^−5^	0.002	3.5x10^−10^			
	**CL**						
**OR**	0.77	0.91	0.79	1.07			
**95% CI**	0.34–1.52	0.41–1.79	0.35–1.55	0.48–2.05			
**p-value**	0.535	1	0.637	0.729			
	**CP**						
**OR**	2.3	2.72	2.35	3.18			
**95% CI**	1.57–3.31	1.86–3.9	1.61–3.37	2.26–4.37			
**p-value**	1.2x10^−5^	1.2x10^−7^	5.8x10^−6^	6x10^−12^			
	**CLP**						
**OR**	1.33	1.57	1.36	1.84			
**95% CI**	0.82–2.08	0.97–2.45	0.84–2.12	1.67–2.76			
**p-value**	0.21	0.056	0.17	1.6 × 10^−6^			

### Neurodevelopmental CNVs and development trajectories

**Figure 1:**
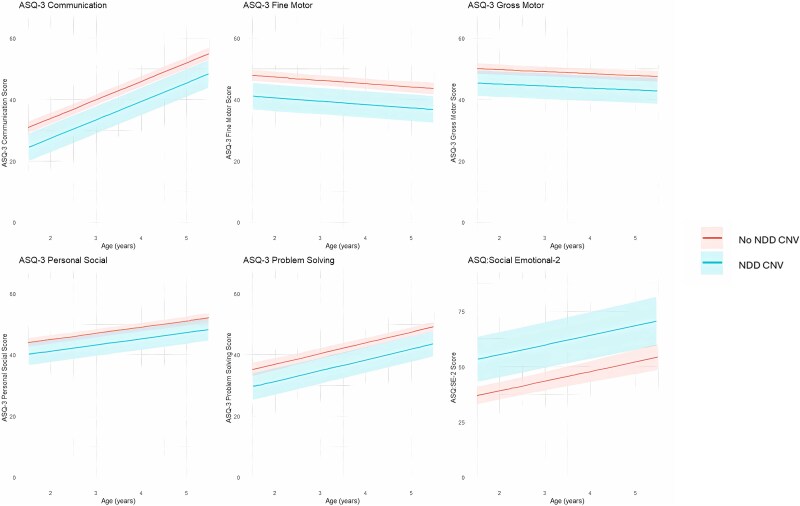
The best-fitting model of predicted scores and 95% confidence intervals from linear multilevel models of ages and stages questionnaire (ASQ)-3 and ASQ:SE-2 scores from ages 18 months to 5 years. Plotted average scores are for a male with a cleft palate both with (blue) and without (red) neurodevelopmental copy number variants (neurodevelopmental CNVs). Please note higher scores on the ASQ-3 domains indicate better developmental outcomes and higher scores on ASQ:SE-2 indicate poorer social emotional outcomes.

To assess early development in children born with CL/P, we used two questionnaires focusing on motor/communication development and emotional development. The Ages and Stages Questionnaire (ASQ-3) [37] assessing early life motor and communication development and the Ages and Stages Questionnaire—Social Emotional (ASQ:SE-2) [38] assessing early life social and emotional development were both completed by mothers when their children were at ages 18 months, 3 years, and 5 years. We calculated mean developmental trajectories of each domain in the ASQ-3 and the ASQ:SE-2 between 18 months to 5 years by fitting a series of linear multilevel models. Starting with a null model for each subscale we incrementally included random intercepts and slopes, sex, the interaction between age and sex, CNV status and cleft sub-type as covariates testing how each term impacted the fit of the data to the model. The terms that were included for each subscale are listed in Table S3. This analysis supports the hypothesis that people born with CL/P and a neurodevelopmental CNV are at higher risk of developmental delays, see Fig. 1, than their peers born with CL/P alone (H3). In linear multilevel models of the ASQ-3 subscales, developmental trajectories of children with neurodevelopmental CNVs were estimated to be consistent with the developmental deficit hypothesis [39]. In line with this hypothesis, developmental delays manifested early in life (lower intercept relative to peers without neurodevelopmental CNVs) but the rate of development (slope of trajectories) was the same as in peers without neurodevelopmental CNVs see Fig. 1 and Table S3. However, children with neurodevelopmental CNVs did not catch up with their peers without CNVs by the age of 5 years.

Each ASQ:SE-2 score from 18 months through to 5 years indicated more developmental impairment across all domains among those with any CL/P and neurodevelopmental CNVs. Those with a neurodevelopmental CNV were estimated to score 16.41 points higher (higher scores on the ASQ:SE-2 indicate higher risk of social–emotional impairment) on the ASQ:SE-2 total score (95%CI 7.05, 26.98, *P* = 4x10^−11^) at 18 months and this persisted through development to age 5 indicating a stable trend of worse social–emotional development relative to peers with CL/P who do not have an neurodevelopmental CNV (Table 3).

**Table 3:** Fixed effect estimates of having a neurodevelopmental copy number variant (ND CNV) in linear multilevel models of the ages and stages questionnaire (ASQ-3) and the ages and stages questionnaire—Social emotional (ASQ:SE-2) in children born with a CL/P. The reference category is no ND CNV. Higher scores on the ASQ-3 domains indicate better developmental outcomes and higher scores on ASQ:SE-2 indicate poorer social–emotional outcomes. Where parameters improved model fit, models are adjusted for sex, interaction with age and sex and cleft sub-type.

Development 18 m to 5 years	ND CNV *N*	No ND CNV *N*	β—Estimate	95% Confidence Intervals	P-value
ASQ-3 Communication	38	941	−6.52	−10.83, −2.22	0.003
ASQ-3 Fine Motor	38	938	−6.80	−10.98, −2.62	0.001
ASQ-3 Gross Motor	38	940	−4.71	−8.76, −0.65	0.023
ASQ-3 Personal Social	38	939	−3.80	−7.25, −0.35	0.031
ASQ-3 Problem Solving	38	939	−5.56	−9.58, −1.54	0.007
ASQ: Social Emotional-2	37	930	16.41	6.52, 26.29	0.001

Cleft palate only, compared to cleft lip only, was associated with poorer outcomes across all ASQ-3 developmental domains at each time point (see Table S4). Children with submucous cleft palate also showed larger negative effects across ASQ-3 domains compared to children with cleft lip only, though confidence intervals were wide due to the small sample size of children with submucous cleft palate. Similarly, ASQ:SE-2 scores indicated more social–emotional problems for children with CP only and submucous cleft palate compared to those with cleft lip only, though wide confidence intervals were also noted for the submucous cleft palate group.

### Neurodevelopmental CNVs and behavioural outcomes

We tested if children born with CL/P and neurodevelopmental CNVs were at higher risk of behavioral problems at age 5 as indicated by the Strengths and Difficulties Questionnaire (SDQ) [40] compared to children with CL/P alone by using linear regression models. Children with CL/P and a neurodevelopmental CNV were estimated to score 4.21 points higher (95%CI 1.48, 6.94, *P* = 0.003) on the SDQ total difficulties scale than their peers with CL/P and no neurodevelopmental CNV. At age 5, children with CL/P and neurodevelopmental CNVs scored higher on average across conduct, emotional, hyperactivity and peer problem subscales and lower on average on the prosocial subscale, all indicating a larger proportion of individuals with higher rates of behavioral difficulties in this group relative to peers with CL/P and no neurodevelopmental CNV, see Table 4. Pooled estimates from imputed data were similar to estimates from the complete cases analyses reported in Table S5.

**Table 4:** Estimated pooled effect of having a neurodevelopmental disorder copy number variant (ND CNV) in linear regression models of the strengths and difficulties questionnaire (SDQ) at age 5. The reference category is no ND CNV. Higher scores on the SDQ indicate higher risk of behavioural problems except for the prosocial subscale where higher scores indicate more prosocial traits. Multiple imputation was used to manage missing data in the SDQ.

*SDQ domains at 5 years*	ND CNV N	No ND CNV N	Pooled effect	95% Confidence Interval	P-value
Conduct	39	959	1.00	(0.24, 1.77)	0.01
Emotional	39	959	0.92	(0.13, 1.72)	0.023
Hyperactivity	39	959	0.52	(−0.63, 1.68)	0.414
Peer Problems	39	959	1.13	(0.38, 1.87)	0.003
Prosocial	39	959	−1.89	(−2.85, −0.93)	0.0001
SDQ Total	39	959	4.21	(1.48, 6.94)	0.0025

SDQ analyses by cleft subtype did not indicate any evidence of differences in behavioural problems by cleft subtype, although sample sizes for this analysis were very limited (see Table S6).

### Individual neurodevelopmental CNV loci

Table 5 summarizes the frequencies of specific neurodevelopmental CNV loci identified > 10 times in children with CL/P compared to the four general population comparison groups we used, with details on all the CNV loci identified in Table S7. To protect anonymity and comply with ethics regulations, individual loci in each cohort where N < 5 are not shown. Three loci—15q11.2, 22q11.2, and 16p11.2—accounted for 73% of the 77 neurodevelopmental CNVs identified. The rates of 15q11.2 deletions and duplications were similar to estimates from the general population, while the 22q11.2 deletion rates were, as expected, elevated in the Cleft Collective (22q11 deletion syndrome is associated with higher risk of cleft palate [26]). We also found a higher proportion of 16p11.2 deletions (0.46%) among children with CL/P, despite this CNV being extremely rare in the general population (<0.03% in UK Biobank and < 5 cases in the other cohorts).

**Table 5:** Neurodevelopmental copy number variation in specific loci among children with cleft vs general population samples. The 4 loci included are those where 10 or more children were identified with that neurodevelopmental CNV among children in the cleft collective. To protect anonymity and comply with ethics regulations, individual loci in each cohort where N < 5 are not shown*.*

		**15q11.2 del BP1-BP2**				**15q11.2 dup BP1-BP2**				**16p11.2 del**				**22q11.2 del**			
		**N (%)**	**OR**	**95% CI**	**P-value**	**N (%)**	**OR**	**95% CI**	**P-value**	**N (%)**	**OR**	**95% CI**	**P-value**	**N (%)**	**OR**	**95% CI**	**P-value**
	**Cleft Collective (N = 2180)**	17 (0.78)				13 (0.60)				10 (0.46)			10 (0.46)				
**Reference Categories**	**ALSPAC (N = 6217)**	42 (0.68)	1.16	0.62, 2.08	0.655	32 (0.51)	1.16	0.56, 2.28	0.613	<5	-	-	-	<5	-	-	-
	**BiB (N = 7477)**	29 (0.39)	2.02	1.04, 3.81	0.032	44 (0.59)	1.01	0.50, 1.92	1	<5	-	-	-	<5	-	-	-
	**MCS (N = 6559)**	52 (0.79)	0.98	0.53, 1.73	1	38 (0.58)	0.92	0.45, 1.77	0.875	<5	-	-	-	<5	-	-	-
	**UK Biobank (N = 151 619)**	544 (0.36)	2.18	1.26, 3.54	0.004	762 (0.50)	1.19	0.63, 2.05	0.539	43 (0.03)	16.24	7.27, 32.91	4.7x10^−13^	5 (0.00)	139.29	43.46529.08	9.8x10^−15^

### Neurodevelopmental CNVs in relatives of children with cleft

We derived neurodevelopmental CNVs in parents and siblings of children from the Cleft Collective. Neurodevelopmental CNVs were present in 2.3% of mothers (54/2344), 3.0% of fathers (54/1869), and 3.4% of siblings (5/144) of children born with CL/P. These rates were similar to those found in general population comparison groups (ALSPAC, BiB, MCS) (Table S8). There was some evidence that parents of children with CL/P had higher odds of having a neurodevelopmental CNV compared to adults in the UK Biobank but not compared to other general population comparison groups (see Table S8).

### Inherited vs *de novo* neurodevelopmental CNVs

We determined if CNVs in children from the Cleft Collective were inherited or *de novo* where parental CNV data were available. Of the 77 children with neurodevelopmental CNVs, 31 (40%) inherited them from a parent, 19 (25%) were *de novo*, and 27 (35%) had an unknown origin. Out of the 27 Individuals with an NDD CNV of unknown origin, 12 had neither parent present in the genotypic data, 8 had only their mother genotyped, 6 had only their father genotyped, and one had both parents whose genotypic data failed QC checks. We investigated whether there were any differences in ASQ-3 and ASQ:SE-2 scores by whether the CNVs arose *de novo* in the child or whether they were inherited from the parents. Whilst there was little to no evidence that *de novo or inherited* neurodevelopmental CNVs impacted early development, there were strong negative associations with developmental outcomes for children who did not have both parents provide genetic data, hence had unknown origin of their neurodevelopmental CNV, see Table S9. Due to small sample sizes, we were not able to test for differences in SDQ scores by inherited or *de novo* CNV status.

## Discussion

This is the first study to investigate and to identify that neurodevelopmental CNVs are more common among children born with CL/P (3.7%) than in the general population (~ 2.2%) (H1). Further, we identified that neurodevelopmental CNVs are most common among the subgroup of children born with a CP only (H2). Making use of longitudinal data we observed that neurodevelopmental CNVs are associated with early development deficits and behavioural problems among children born with CL/P from 18 months to 5 years of age (H3). In addition, we observed that along with 22q11, 16p11.2 appears to be a specific CNV locus relevant to CP.

Children with CL/P are more likely to have neurodevelopmental CNVs compared to the general population, and the evidence indicates that those with both CL/P and neurodevelopmental CNVs face a higher risk of behavioral problems and developmental delays than their peers with CL/P but without neurodevelopmental CNVs. These findings suggest that neurodevelopmental CNVs may have pleiotropic effects, influencing both the development of CL/P and behavioural differences. Alternatively, children with CL/P and neurodevelopmental CNVs may have more complex medical needs and/or be more likely to be exposed to adverse experiences, such bullying and discrimination, compared to their peers with CL/P but no neurodevelopmental CNVs which in turn could be increasing behavioural symptoms. Regardless of the specific causal pathway, CNVs that increase risk to neurodevelopmental disorders are observed in higher rates in children born with CL/P compared to children from the general population and this could be linked to their higher rates of behavioural problems and developmental delays. However, the majority of children with a neurodevelopmental CNV in the Cleft Collective (68%) were not recorded as having a syndromic form of CL/P (from parent or surgeon reports). This suggests that the majority of parents are probably unaware of their child’s CNV status and the health implications this might have currently or in the future.

Children born with CP only were more likely to have neurodevelopmental CNVs and developmental delays compared to children born with cleft lip only. This is consistent with previous research on CP indicating higher risk of having a syndromic cleft amongst those with CP only. In addition, genome-wide association studies and epigenome-wide association studies have identified CP as a phenotype with distinct aetiology from cleft lip and other cleft phenotypes [4, 5, 41].

Rates of deletions in the 16p11.2 locus were unusually high among the Cleft Collective children; 10 in 2180 (0.46%) as compared to general population estimates of 3 in 10 000 (43/151619, 0.03%) in UK Biobank and less than five cases in ALSPAC, BiB and MCS. Recent work on 16p11.2 deletion syndrome suggests that a minor proportion of those affected present with small craniofacial malformations, among which cleft is mentioned, but the core distinguishing features for the majority with this neurodevelopmental CNV are developmental delays in language, motor difficulties, autism, seizures and obesity in adulthood [19, 25, 42, 43]. Communication and language are also commonly delayed across 15q.11.2 [44] and 22q.11.2 [26] deletion syndromes.

The interpretation of impaired communication among children born with CL/P is not straightforward. Low scores in the communication subscale may represent a causal effect of CL/P [45] and not a direct genetic effect, i.e. children born with CLP or CP have a higher risk of speech disorder which in turn can impact language and communication development. Nevertheless, those with neurodevelopmental CNVs and CL/P did consistently score lower than their peers in the Cleft Collective on communication subscales, perhaps indicative of a ‘double hit’ on the development of communication among those with CL/P and neurodevelopmental CNVs.

Recent longitudinal research has described the development of children with non-syndromic CL/P as delayed on each of the ASQ-3 subscales relative to typically developing children and differences emerged between 18 months and 2 years [10]. In the present study, those with CL/P and neurodevelopmental CNVs showed early deficits on the ASQ-3 compared to those with CL/P but without neurodevelopmental CNVs, although the rate of development was the same in both groups. These differences were stable and persisted from 18 months through to 5 years across each subscale in line with the developmental deficit hypothesis [39]. According to this hypothesis, impaired development manifests early in life. Despite the rate of development being the same as in typically developing children, children with CL/P and neurodevelopmental CNVs do not catch up from this early developmental deficit. Thus, while those without neurodevelopmental CNVs may also be at risk of delayed development, our data suggest that the vulnerability of those with CL/P and neurodevelopmental CNVs is greater. Findings from a study of children with 22q11.2 deletion syndrome, which has cleft palate as one of its associated features, and unaffected siblings also supported the developmental deficits hypothesis. There was little evidence of deterioration in trajectories of cognitive development in children with 22q11.2 deletions despite the early deficits compared to unaffected siblings [46].

Given that a proportion of neurodevelopmental CNVs are inherited, it is possible that indirect genetic effects may play a role i.e. that parental genetic architecture impacts parenting resources and the child’s early environment hence later development. However, in this study, we did not find evidence to suggest that inherited or *de novo* CNVs had a greater impact on outcomes. Rather, we found that the ‘unknown’ category had strong evidence of association with early development and behavioural outcomes. This suggests that having two parents participate and provide genetic data seems protective for developmental delay. In other words, selection bias into the study had a stronger effect than whether a neurodevelopmental CNV was inherited or *de novo*.

### Strengths and limitations

The main challenge of examining cleft, a rare outcome, in the context of rare genetic features (CNVs), is having a sufficient sample size to detect effects. The Cleft Collective is the largest longitudinal dataset of children with CL/P with genotypes available on children and their parents. The effects reported in this study are sufficiently large that even where samples sizes are limited, we were able to observe strong associations. This serves to support the overall conclusion that even with small numbers we can observe that neurodevelopmental CNVs are more common among children with cleft and have a notable impact on both development and behavioural problems.

Making use of four different general population samples each with their own different selection biases [47] and consistently estimating similar results across these samples adds strength to the confidence we can have in our results. Specifically, ALSPAC, BiB and MCS are birth cohorts and comparable in terms of data collection methodology and timings to the Cleft Collective, which for genetic data was early in life. UK Biobank provides a much larger sample, but recruited 40–69-year-olds and hence estimates of neurodevelopmental CNVs are anticipated, and are observed, to be lower due to survival bias. However, this large sample allows for comparisons even for rare outcomes, such as 16p.11.2 deletions.

Selection bias and attrition are important concerns in any longitudinal study and they are associated with genetic liability to neurodevelopmental and psychiatric phenotypes [48, 49]. We observed selection bias in the Cleft Collective with strong associations of parents not providing DNA with lower developmental outcomes for children. This suggests that we might be underestimating the rates of neurodevelopmental CNVs in the Cleft Collective if parents of children with neurodevelopmental concerns and/or CNVs are less likely to participate.

We note that we only examined one type of rare genetic mutations. Common variants have long been established to be associated with CL/P [4, 50] and neurodevelopmental disorders [51–53] and examining whether they contribute to behavioral problems in children born with CL/P is equally important.

There are several statistical tests performed in this analysis which does increase the chance of type 1 error, however, we present confidence intervals around all our effect estimates and urge the reader to infer the robustness of an effect estimate using these values as opposed to applying a multiple testing correction and shifting arbitrary p-value thresholds [54, 55].

Clinical diagnoses of specific neurodevelopmental disorders were not assessed in this study. Instead, we relied on scores measured by scales, such as SDQ and ASQ which are strongly predictive of developmental and behavioural problems [38, 40, 56]. The study population would be too young by age 5, to have received a diagnosis of a childhood neurodevelopmental or psychiatric disorder, hence it is not appropriate to use categorical measures. Further, the use of continuous scores maximises variance in the data to provide greater statistical power. The early indicators of developmental and behavioural problems that we identified do however provide a strong rationale for continued follow up in the Cleft Collective and/or linkage with primary and secondary care studies.

### Implications

The evidence set out in this paper suggests children born with CL/P are at higher risk of neurodevelopmental CNVs (3.7% vs 2.2% in the general population), and children with neurodevelopmental CNVs and CL/P are at higher risk of behavioural problems at 5 years and developmental delays before the age of 5 years. The implication is that genetic testing to ascertain the presence of neurodevelopmental CNVs might be helpful in early identification of developmental needs in children born with CL/P, as well as signposting the need for follow up and early interventions [57] to reduce the impact as children grow. This is particularly important because the majority of parents whose child had a CNV did not report being aware that they might have a syndromic form of cleft. One of the strongest associations noted in this study was the social–emotional development assessed by ASQ:SE-2 scores which include parent-reported concerns about their child. Contextualizing parental concern with possible explanatory power of genetic testing may help support parents understanding their child’s developmental needs. The results in this paper also substantiate the policy among cleft teams of the inclusion of clinical psychologists to support children born with CL/P.

## Conclusion

Children born with CL/P are at greater risk of carrying an neurodevelopmental CNV than the general population, with the highest risk observed in those with CP only. Furthermore, those with both an neurodevelopmental CNV and CL/P experience an elevated risk of early developmental delays from 18 months to 5 years and are more likely to experience behavioural problems by age 5. These findings underscore the importance of early genetic screening and tailored interventions to address the unique developmental challenges faced by this group. Follow-up of children’s developmental and behavioural traits as they grow up as well as linkage to their educational attainment data could reveal further consequences of neurodevelopmental CNVs in children born with CL/P.

## Materials and methods

### Cleft collective

The Cleft Collective [28] is an ongoing UK-based national cohort study of children born with CL/P, their parents, and siblings. Details of recruitment and data collection procedures can be found elsewhere [8, 9] along with how to access the resource at https://www.bristol.ac.uk/cleft-collective/professionals/access/. Briefly, data are collected from two cohorts, the birth cohort and a cohort of five-year olds. Families are recruited to the birth cohort during pregnancy (if cleft is detected during ultrasound scans) or soon after birth but before the study child’s primary surgery to repair their cleft. Families are recruited to the five-year cohort between the child’s fifth and sixth birthday, often at a five-year follow-up clinic. Data used to address the outlined hypotheses are from a combination of parental questionnaires and biological samples collected by families or medical professionals. All study participants were recruited with informed parental consent. The Cleft Collective Cohort Studies received ethical approval from the South-West Central Bristol NRES Ethics Committee (REC 13/SW/0064). This project was approved by the Cleft Collective project management group (CC048-ES).

### Genotyping

Study children from the birth cohort provided blood and/or tissue samples, consenting parents and siblings from both cohorts and study children from the 5-year-old cohort provided saliva samples. These were all genotyped in the Illumina facility in Bristol Bioresource Laboratories, Bristol UK, using the Illumina Global Screening Array (GSA) version 3. Raw data from 7182 samples were uploaded to GenomeStudio where we carried out preliminary Quality Control (QC) processes in which 264 samples were removed, see Fig. S1. We exported log R ratios and B-allele frequencies for each of the remaining 6918 samples to identify CNVs.

### CNV calling

We limited the CNVs called to a pre-determined list of 54 CNVs that have been associated with neurodevelopmental disorders [29], the full list is reported in Table S2. We adapted the following neurodevelopmental CNV calling pipeline: https://github.com/CardiffMRCPathfinder/NeurodevelopmentalCNVCalling.git [58] using PennCNV 1.0.5 [59]. This is described in detail in the Supplementary Note 1 and Fig. S2. Briefly, we performed initial QC excluding samples based on 100 or more CNVs, waviness factor < −0.037 or > 0.037, or log R ratio SD > 0.24. From 7182 raw samples, the final sample consisted of 6551, see Figs S1, S3 and S4. We visually inspected log R ratios and B-allele frequencies of each NDD CNV to exclude low quality calls, see Fig. S5 for examples of CNV calls.

Where data were available for a study child and both parents, we were able to determine if a neurodevelopmental CNV was inherited or *de novo*. Where genetic data from either parent were missing, inherited/*de novo* status remained unknown.

### Neurodevelopmental CNV control samples

We included published rates of neurodevelopmental CNVs in children from the Avon Longitudinal Study of Parents and Children (ALSPAC) [60], Born in Bradford (BiB) [33], the Millenium Cohort Study (MCS) [60] and adult data from UK Biobank [36] to provide a general population baseline for neurodevelopmental CNVs. ALSPAC is a long-standing multi-generational cohort, recruited in the early 1990s from prospective mothers in the county of Avon and has multiple waves of rich phenotypic information on them and their offspring [30, 31]. BiB was established in Bradford in 2007, recruiting prospective mothers, and has notable ethnic diversity compared with other UK-based cohorts [32]. MCS is a nation-wide cohort from children born in 2000–2001, with intentional oversampling of areas with ethnic minorities as well as deprivation [34]. UK Biobank is a large-scale database of over 500 000 UK adults providing extensive genetic, lifestyle, and health information [35].

For each cohort the same set of 54 neurodevelopmental CNVs were identified and the same pipeline used to perform the calls [29].

### Measures

#### Demographics

We estimated sex from genetic data using the ‘sex estimate’ function in GenomeStudio2.0. Maternal ethnicity and educational attainment were collected via baseline questionnaires. To ensure participant anonymity and comply with our ethical approval which stipulates cell counts ≥5, six ethnic groups were collapsed to two, ‘white’ and ‘other’. Maternal education categories were collapsed from 14 options into three groups, ‘None/fewer than 5 GCSEs or equivalent’, ‘Post-Secondary Education (Non-degree)’ and ‘Higher Education (Degree or equivalent)’.

### Cleft sub-type

Cleft sub-type (cleft lip, cleft palate, unilateral cleft lip and palate, bilateral cleft lip and palate, and submucous palate) was derived from parent-reported and surgeon-reported questionnaires. Due to low cell counts, we combined the ‘unilateral’ and ‘bilateral’ categories to form a new category ‘cleft lip and palate’. Parent/surgeon-reported syndrome was derived from a binary variable from surgeon and/or parental questionnaires.

### Neurodevelopmental and behavioural problem measures

All measures below were administered in the form of questionnaires completed by both mothers and fathers.

#### Developmental delays

The Ages and Stages Questionnaire (ASQ-3) [37] was used to assess development across five domains: Communication, Gross Motor, Fine Motor, Problem Solving, and Personal-Social. Each domain is evaluated through six items, where responses are categorised as ‘yes’ (10 points), ‘sometimes’ (5 points), or ‘not yet’ (0 points). Scores for each domain range from 0 to 60, with higher scores indicating more advanced development. In cases where up to two items were missing within a domain, these missing answers were replaced with the mean of the items that were answered. The ASQ-3 was administered at ages 18 months, 3 years, and 5 years. For reference, the cut offs for each ASQ measure which indicate clinical monitoring are outlined in Table S10.

#### Social and emotional development

Social and emotional development was measured using the Ages and Stages Questionnaire—Social Emotional (ASQ:SE-2) [38]. Higher scores reflect poorer social–emotional functioning. The scores are treated as continuous variables for analysis. The ASQ:SE-2 was collected at ages 18 months, 3 years, and 5 years. For reference, the cut-offs for each ASQ measure which indicate clinical monitoring or referral are outlined in Table S10. The possible score range for each age is as follows: 18 months; 0–360, 3 years; 0–465, 5 years; 0–405.

#### Behavioural problems

The Strengths and Difficulties Questionnaire (SDQ) [40], administered at age 5, was used to assess behavioural problems. This 25-item screening tool covers five subscales: conduct problems, emotional symptoms, hyperactivity, peer problems, and prosocial behaviour. There are 5 items on each subscale and options for each item are ‘not true’ coded as zero, ‘somewhat true’ coded as one and ‘certainly true’ coded as two. Higher scores on all subscales, except prosocial behaviour, indicate a higher risk of behavioural issues. Four subscales (conduct problems, emotional symptoms, hyperactivity, peer problems) combine to form a total difficulties score, with a score of 17 or above flagging high risk. High-risk cut-offs for detailed assessment are: conduct problems (four or more), emotional symptoms (five or more), hyperactivity (six or more), peer problems (seven or more), and prosocial behaviour (less than four) [61].

For each of the neurodevelopmental and behavioural measures described above we used continuous scores to maximise variance in the data [56]. Due to a higher response rate, maternal responses were the default score used; where maternal data were unavailable, we used paternal data to complete scores. Correlations between the equivalent scores from maternal and paternal raters were positive and high across all SDQ measures, low to moderate for ASQ:SE-2 and moderate to high for all ASQ-3 measures, see Figs S6 and S7.

### Statistical analysis

To test H1, we generated frequency tables with counts of Cleft Collective study children with and without neurodevelopmental CNVs, and the equivalent reference data from four general population comparison groups: Avon Longitudinal Study of Children and Parents (ALSPAC), Millenium Cohort Study (MCS), Born in Bradford (BiB) and UK Biobank. We calculated odds ratios (ORs), confidence intervals, and p-values using Fischer’s exact test. We repeated these analyses stratified by cleft type (H2). To further assess the prevalence of neurodevelopmental CNVs by cleft type we performed logistic regression with presence of neurodevelopmental CNVs as an outcome, cleft sub-type as a categorical predictor and sex as a covariate.

### Multilevel models

To determine the best fit of mean development of each domain in the ASQ-3 and the ASQ:SE-2 across the three time points, between 18 months to 5 years, we fitted a series of linear multilevel models increasing in complexity from null models to random intercepts and random slopes. We incrementally included sex, the interaction between age and sex, and cleft sub-type as covariates. The interaction term was included to model how sex-specific trajectories change over time. Covariates were included in the model if they improved model fit by reducing Akaike Information Criterion (AIC) and showing a Loglikelihood Ratio (LR) tests at *P* < 0.05. Age was centered at 18 months. The fit statistics and final formula for each model are presented in Table S3.

To test H3, we included the presence or absence of an neurodevelopmental CNV as a level-1 predictor in each multilevel model. We also tested for any interactions between neurodevelopmental CNVs and age to assess if there were changes in the impact of neurodevelopmental CNVs over time. To assess if subgroups of children with neurodevelopmental CNVs develop differently we included ‘cleft sub-type’ and ‘inherited vs de novo’ as additional analyses. All multilevel models were estimated using ‘R2MLwiN’ in R and ‘MLwiN’ [62, 63].

To further test H3 in a cross-sectional framework and estimate associations between the presence of a neurodevelopmental CNV and SDQ scores at age 5 we used linear regression models. In each regression model we included sex and age as covariates, we retained the covariates where LR test at P < 0.05 indicated improved fit. All statistical analyses were performed in R 4.3.2.

### Missing data

We used multiple imputation to manage missing data in linear regression models. The imputation model was limited to those who had at least one outcome measure (ASQ-3, ASQ:SE-2 or SDQ) present at one time point. We included all outcome measures at each time point (ASQ-3, ASQ:SE-2, SDQ) and all demographic measures listed above. We used the package ‘mice’ [64] in R and having established the percentage of missing data was ~ 30%, generated 30 complete datasets through 10 iterations of predictive mean matching. We report the overall pooled estimates for each regression. For comparison, complete cases models are also reported in Table S5.

## Supplementary Material

CNV_Cleft_Supplementary_Materials_ddaf115

## Notes

MJO reports a grant from Akrivia Health outside of the submitted work.

ER reports receiving grants from Akrivia Health outside the submitted work.

## References

[1] Leslie EJ, Marazita ML. Genetics of cleft lip and cleft palate. Am J Med Genet C Semin Med Genet 2013;163C:246–258. 24124047 10.1002/ajmg.c.31381 PMC3925974

[2] Carlson JC, Anand D, Butali A. et al. A systematic genetic analysis and visualization of phenotypic heterogeneity among orofacial cleft GWAS signals. Genet Epidemiol 2019;43:704–716. 31172578 10.1002/gepi.22214 PMC6687557

[3] Babai A, Irving M. Orofacial clefts: genetics of cleft lip and palate. Genes (Basel) 2023;14:1603. 10.3390/genes14081603 PMC10454293 37628654

[4] Robinson K, Mosley TJ, Rivera-González KS. et al. Trio-based GWAS identifies novel associations and subtype-specific risk factors for cleft palate. HGG Adv 2023;4:100234. 37719664 10.1016/j.xhgg.2023.100234 PMC10502411

[5] Sharp GC, Ho K, Davies A. et al. Distinct DNA methylation profiles in subtypes of orofacial cleft. Clin Epigenetics 2017;9:63. 28603561 10.1186/s13148-017-0362-2 PMC5465456

[6] Robinson K, Curtis SW, Leslie EJ. The heterogeneous genetic architectures of orofacial clefts. Trends Genet 2024;40:410–421. 38480105 10.1016/j.tig.2024.02.004

[7] Lockhart E . The mental health needs of children and adolescents with cleft lip and/or palate. Clin Child Psychol Psychiatry 2003;8:7–16.

[8] Stock NM, Feragen KB. Psychological adjustment to cleft lip and/or palate: a narrative review of the literature. Psychol Health 2016;31:777–813. 26800428 10.1080/08870446.2016.1143944

[9] Berman S, Sharp GC, Lewis SJ. et al. Prevalence and factors associated with Behavioral problems in 5-year-old children born with cleft lip and/or palate from the cleft collective. Cleft Palate Craniofac J 2024;61:40–51. 36083151 10.1177/10556656221119684 PMC10676624

[10] Tsuchiya S, Tsuchiya M, Momma H. et al. Neurodevelopmental trajectories in children with cleft lip and palate: a longitudinal study based on the Japan environment and Children's study. Eur J Oral Sci 2022;130:e12857. 35166390 10.1111/eos.12857

[11] Dardani C, Howe LJ, Mukhopadhyay N. et al. Cleft lip/palate and educational attainment: cause, consequence or correlation? A mendelian randomization study. Int J Epidemiol 2020;49:1282–1293. 32373937 10.1093/ije/dyaa047 PMC7660147

[12] Wehby GL, Collett BR, Barron S. et al. Children with oral clefts are at greater risk for persistent low achievement in school than classmates. Arch Dis Child 2015;100:1148–1154. 26347387 10.1136/archdischild-2015-308358 PMC5018039

[13] Branson EK, Branson VM, McGrath R. et al. Psychological and peer difficulties of children with cleft lip and/or palate: a systematic review and meta-analysis. Cleft Palate Craniofac J 2024;61:258–270. 36082954 10.1177/10556656221125377

[14] Stock NM, Costa B, White P. et al. Risk and protective factors for psychological distress in families following a diagnosis of cleft lip and/or palate. Cleft Palate Craniofac J 2020;57:88–98. 31378083 10.1177/1055665619862457

[15] Conte F, Oti M, Dixon J. et al. Systematic analysis of copy number variants of a large cohort of orofacial cleft patients identifies candidate genes for orofacial clefts. Hum Genet 2016;135:41–59. 26561393 10.1007/s00439-015-1606-x PMC4698300

[16] Cai Y, Patterson KE, Reinier F. et al. Copy number changes identified using whole exome sequencing in nonsyndromic cleft lip and palate in a Honduran population. Birth Defects Res 2017;109:1257–1267. 28748635 10.1002/bdr2.1063 PMC5854563

[17] Lansdon LA, Dickinson A, Arlis S. et al. Genome-wide analysis of copy-number variation in humans with cleft lip and/or cleft palate identifies COBLL1, RIC1, and ARHGEF38 as clefting genes. Am J Hum Genet 2023;110:71–91. 36493769 10.1016/j.ajhg.2022.11.012 PMC9892779

[18] Cunningham AC, Hall J, Einfeld S. et al. Assessment of emotions and behaviour by the developmental behaviour checklist in young people with neurodevelopmental CNVs. Psychol Med 2022;52:574–586. 32643597 10.1017/S0033291720002330 PMC7794095

[19] Gur RC, Bearden CE, Jacquemont S. et al. Neurocognitive profiles of 22q11.2 and 16p11.2 deletions and duplications. Mol Psychiatry 2025;30:379–387. 39048645 10.1038/s41380-024-02661-y PMC11746132

[20] Williams NM, Franke B, Mick E. et al. Genome-wide analysis of copy number variants in attention deficit hyperactivity disorder: the role of rare variants and duplications at 15q13.3. Am J Psychiatry 2012;169:195–204. 22420048 10.1176/appi.ajp.2011.11060822 PMC3601405

[21] Rees E, Walters JT, Georgieva L. et al. Analysis of copy number variations at 15 schizophrenia-associated loci. Br J Psychiatry 2014;204:108–114. 24311552 10.1192/bjp.bp.113.131052 PMC3909838

[22] Leppa VM, Kravitz SN, Martin CL. et al. Rare inherited and De novo CNVs reveal complex contributions to ASD risk in multiplex families. Am J Hum Genet 2016;99:540–554. 27569545 10.1016/j.ajhg.2016.06.036 PMC5011063

[23] Chawner S, Owen MJ, Holmans P. et al. Genotype-phenotype associations in children with copy number variants associated with high neuropsychiatric risk in the UK (IMAGINE-ID): a case-control cohort study. Lancet Psychiatry 2019;6:493–505. 31056457 10.1016/S2215-0366(19)30123-3

[24] Chawner S, Doherty JL, Anney RJL. et al. A genetics-first approach to dissecting the heterogeneity of autism: phenotypic comparison of autism risk copy number variants. Am J Psychiatry 2021;178:77–86. 33384013 10.1176/appi.ajp.2020.20010015 PMC8022239

[25] Ali NMH, Chawner S, Kushan-Wells L. et al. Comparison of autism domains across thirty rare variant genotypes. EBioMedicine 2025;112:105521. 39891993 10.1016/j.ebiom.2024.105521 PMC11835590

[26] Cortes-Martin J, Penuela NL, Sanchez-Garcia JC. et al. Deletion syndrome 22q11.2: a systematic review. Children (Basel) 2022;9:1168. 10.3390/children9081168 PMC9406687 36010058

[27] Costa B, White P, Kiff JD. et al. Parent-reported socioemotional and cognitive development in children with a cleft lip and/or palate at 18 months: findings from a UK birth cohort. Child Care Health Dev 2021;47:31–39. 32990944 10.1111/cch.12813

[28] Davies AJV, Humphries K, Lewis SJ. et al. The cleft collective: protocol for a longitudinal prospective cohort study. BMJ Open 2024;14:e084737. 10.1136/bmjopen-2024-084737 PMC11227803 38969383

[29] Kendall KM, Rees E, Bracher-Smith M. et al. Association of Rare Copy Number Variants with risk of depression. JAMA Psychiatry 2019;76:818–825. 30994872 10.1001/jamapsychiatry.2019.0566 PMC6583866

[30] Boyd A, Golding J, Macleod J. et al. Cohort profile: the ‘children of the 90s’—the index offspring of the Avon longitudinal study of parents and children. Int J Epidemiol 2013;42:111–127. 22507743 10.1093/ije/dys064 PMC3600618

[31] Fraser A, Macdonald-Wallis C, Tilling K. et al. Cohort profile: the Avon longitudinal study of parents and children: ALSPAC mothers cohort. Int J Epidemiol 2013;42:97–110. 22507742 10.1093/ije/dys066 PMC3600619

[32] Wright J, Small N, Raynor P. et al. Cohort profile: the born in Bradford multi-ethnic family cohort study. Int J Epidemiol 2013;42:978–991. 23064411 10.1093/ije/dys112

[33] Ali N . Neurodevelopmental and neuropsychiatric disorders in individuals with rare pathogenic genetic variants analysis in clinical and population-based cohorts PhD thesis, Cardiff University, 2023. URL: https://orca.cardiff.ac.uk/id/eprint/164975/1/2023AliN%20PhD.pdf.

[34] Connelly R, Platt L. Cohort profile: UK millennium cohort study (MCS). Int J Epidemiol 2014;43:1719–1725. 24550246 10.1093/ije/dyu001

[35] Sudlow C, Gallacher J, Allen N. et al. UK biobank: an open access resource for identifying the causes of a wide range of complex diseases of middle and old age. PLoS Med 2015;12:e1001779. 25826379 10.1371/journal.pmed.1001779 PMC4380465

[36] Kendall KM, Rees E, Escott-Price V. et al. Cognitive performance among carriers of pathogenic copy number variants: analysis of 152,000 UK biobank subjects. Biol Psychiatry 2017;82:103–110. 27773354 10.1016/j.biopsych.2016.08.014

[37] Squires J . ASQ-3 user's guide. Baltimore: Paul H. Brookes Pub, 2009.

[38] Squires J, Bricker DD, Twombly E. Ages & stages questionnaires, social-emotional (ASQ:SE-2TM): a parent-completed child monitoring system for social-emotional behaviors. Baltimore, Maryland: Brookes Publishing, 2015.

[39] Weinberger DR . Implications of normal brain development for the pathogenesis of schizophrenia. Arch Gen Psychiatry 1987;44:660–669. 3606332 10.1001/archpsyc.1987.01800190080012

[40] Goodman R . Psychometric properties of the strengths and difficulties questionnaire. J Am Acad Child Adolesc Psychiatry 2001;40:1337–1345. 11699809 10.1097/00004583-200111000-00015

[41] Sivertsen A, Wilcox AJ, Skjaerven R. et al. Familial risk of oral clefts by morphological type and severity: population based cohort study of first degree relatives. BMJ 2008;336:432–434. 18250102 10.1136/bmj.39458.563611.AE PMC2249683

[42] Niarchou M, Chawner S, Doherty JL. et al. Psychiatric disorders in children with 16p11.2 deletion and duplication. Transl Psychiatry 2019;9:8. 30664628 10.1038/s41398-018-0339-8 PMC6341088

[43] Chung WK, Roberts TP, Sherr EH. et al. 16p11.2 deletion syndrome. Curr Opin Genet Dev 2021;68:49–56. 33667823 10.1016/j.gde.2021.01.011 PMC10256135

[44] Cox DM, Butler MG. The 15q11.2 BP1-BP2 microdeletion syndrome: a review. Int J Mol Sci 2015;16:4068–4082. 25689425 10.3390/ijms16024068 PMC4346944

[45] van Eeden S, Wren Y, McKean C. et al. Early communication Behaviors in infants with cleft palate with and without Robin sequence: a preliminary study. Cleft Palate Craniofac J 2022;59:984–994. 34259062 10.1177/10556656211031877 PMC9272514

[46] Chawner S, Doherty JL, Moss H. et al. Childhood cognitive development in 22q11.2 deletion syndrome: case-control study. Br J Psychiatry 2017;211:223–230. 28882829 10.1192/bjp.bp.116.195651 PMC5623878

[47] Schoeler T, Speed D, Porcu E. et al. Participation bias in the UK biobank distorts genetic associations and downstream analyses. Nat Hum Behav 2023;7:1216–1227. 37106081 10.1038/s41562-023-01579-9 PMC10365993

[48] Munafo MR, Tilling K, Taylor AE. et al. Collider scope: when selection bias can substantially influence observed associations. Int J Epidemiol 2018;47:226–235. 29040562 10.1093/ije/dyx206 PMC5837306

[49] Taylor AE, Jones HJ, Sallis H. et al. Exploring the association of genetic factors with participation in the Avon longitudinal study of parents and children. Int J Epidemiol 2018;47:1207–1216. 29800128 10.1093/ije/dyy060 PMC6124613

[50] Leslie EJ, Carlson JC, Shaffer JR. et al. A multi-ethnic genome-wide association study identifies novel loci for non-syndromic cleft lip with or without cleft palate on 2p24.2, 17q23 and 19q13. Hum Mol Genet 2016;25:ddw104–ddw2872. 10.1093/hmg/ddw104 PMC5181632 27033726

[51] Grove J, Ripke S, Als TD. et al. Identification of common genetic risk variants for autism spectrum disorder. Nat Genet 2019;51:431–444. 30804558 10.1038/s41588-019-0344-8 PMC6454898

[52] Trubetskoy V, Pardinas AF, Qi T. et al. Mapping genomic loci implicates genes and synaptic biology in schizophrenia. Nature 2022;604:502–508. 35396580 10.1038/s41586-022-04434-5 PMC9392466

[53] Demontis D, Walters GB, Athanasiadis G. et al. Genome-wide analyses of ADHD identify 27 risk loci, refine the genetic architecture and implicate several cognitive domains. Nat Genet 2023;55:198–208. 36702997 10.1038/s41588-022-01285-8 PMC10914347

[54] Sterne JA, Davey Smith G. Sifting the evidence-what's wrong with significance tests? BMJ 2001;322:226–231. 11159626 10.1136/bmj.322.7280.226 PMC1119478

[55] Wasserstein RL, Lazar NA. The ASA statement on p-values: context, process, and purpose. Am Stat 2016;70:129–133.

[56] Goodman A, Goodman R. Strengths and difficulties questionnaire as a dimensional measure of child mental health. J Am Acad Child Adolesc Psychiatry 2009;48:400–403. 19242383 10.1097/CHI.0b013e3181985068

[57] Lane H, Harding S, Wren Y. A systematic review of early speech interventions for children with cleft palate. Int J Lang Commun Disord 2022;57:226–245. 34767284 10.1111/1460-6984.12683

[58] Lynham AJ, Knott S, Underwood JFG. et al. DRAGON-data: a platform and protocol for integrating genomic and phenotypic data across large psychiatric cohorts. BJPsych Open 2023;9:e32. 36752340 10.1192/bjo.2022.636 PMC9970169

[59] Wang K, Li M, Hadley D. et al. PennCNV: an integrated hidden Markov model designed for high-resolution copy number variation detection in whole-genome SNP genotyping data. Genome Res 2007;17:1665–1674. 17921354 10.1101/gr.6861907 PMC2045149

[60] Dennison CA, Martin J, Shakeshaft A. et al. Early manifestations of neurodevelopmental copy number variants in children: a population-based investigation. Biol Psychiatry 2025; in press. 10.1016/j.biopsych.2025.03.004 40090564

[61] Goodman R, Ford T, Simmons H. et al. Using the strengths and difficulties questionnaire (SDQ) to screen for child psychiatric disorders in a community sample. Br J Psychiatry 2000;177:534–539. 11102329 10.1192/bjp.177.6.534

[62] Zhang Z, Parker RMA, Charlton CMJ. et al. R2MLwiN: a package to run MLwiN from within R. J Stat Softw 2016;72:1–43.

[63] Charlton C, Rasbash J, Browne WJ. et al. MLwiN Version 3.14. Centre for Multilevel Modelling. University of Bristol, Bristol, 2025.

[64] van Buuren S, Groothuis-Oudshoorn K. Mice: multivariate imputation by chained equations in R. J Stat Softw 2011;45:1–67.

